# A Case of Bright’s Disease, Etc.

**Published:** 1872-04

**Authors:** Reuben A. Vance

**Affiliations:** New York City


*A Case of Bright's Disease of the Kidneys, in which there was no Albuminuria, the only evidence of Renal Disease being the characteristic alterations of the intra-ocular structures as determined by the Ophthalmoscope.* By REUBEN A. VANCE, M.D., New York City. Reprinted from the Boston Med. and Surgical Journal of March 28, 1872.

It has long been known that certain diseases which implicate different bodily organs may present, as part of their phenomena, symptoms which, when considered alone, would seem to indicate the various forms of cerebral disease. This fact especially has tended to make the diagnosis of cerebro-spinal disorders one of the most obscure and difficult problems in practical medicine. The effect of various poisons introduced into the blood in producing abnormal manifestations of cerebro-spinal phenomena is so common as to have doubtless come under the observation of all my readers, and need not be dwelt upon here. The effects of alcohol, hashisch, opium, etc., are well known; yet the fact that their different stages display in a brief period of time the symptoms and physical manifestations of diseases which are of a different origin, pursue a different course, and are in no way related to the causes which are in action in the former class of cases, is not so well known. The same is true of organic diseases affecting different parts of the body. The delirium of pneumonia and the essential fevers, the exaltation of mind in phthisis, and the melancholia of cancer, are familiar examples.

Among those diseases which primarily implicate organic structure and secondarily modify the quantity or quality of the circulating fluid, none are of more importance than those affections of the kidneys which are ordinarily known as Bright's disease. There is no organ in the body which is not affected by the changes which this disease produces in the blood, and there are few cases which present themselves to the physician in which it is not desirable to determine the presence or absence of this pathological state. In cerebral diseases this is notoriously the case, and it is always necessary, before ascribing any obscure nervous symptom to disease of the organ manifesting it, to be accurately informed as to the state of the kidneys—the presence or absence of albumen in the urine, and the existence or non-existence of nephritic casts.

The ordinary method of determining the existence of kidney disease is by testing the urine. In this way results are obtained which are of the greatest importance in pathology and treatment. Various methods have been devised to aid the practitioner in this task, some of which are cumbersome and some simple and expeditious.* Whatever the plan adopted or the apparatus employed, the object to be attained is simply the determination of the existence of certain abnormal ingredients in the specimen of urine under examination. In the vast majority of cases of kidney disease the urine will reveal evidences which will settle the case in one way or the other. Yet that such is not always so can be seen by the following case, which I have reported at length on account of its close resemblance to well-known forms of brain disease.

Some months since, a gentleman engaged in a wholesale business which had been greatly embarrassed by the Chicago fire, called at my office, complaining of wakefulness, headache, vertigo, and confusion of ideas. He was continually harboring disagreeable thoughts, and could not obtain rest either by day or night. The ideas he entertained relative to the feelings of his friends

* VANCE. Chemical Examination of the Urine, with a description of a convenient Apparatus for its speedy Analysis. *Medical World*, September, 1871.

towards him were grossly erroneous, and although he would admit his mistake when expostulated with by them, yet an hour or two subsequently he would find himself in the same old disagreeable, doubting, distrustful state of mind. His sleep was unsound, and greatly disturbed by dreams of a grotesque rather than a disagreeable nature. Late in the afternoon and during the evening he was subject to vertigo, which would often become so severe as to cause him to fall to the ground. Unable to sleep, he would occasionally walk the streets until quite late, and on one occasion during an attack of vertigo was arrested and taken to the station-house before the officer discovered that he was not dealing with a drunken man. He suffered greatly from depression of spirits, and lost interest in many of his former pursuits. He was conscious of the gravity of these symptoms, and had resorted to a number of measures designed to relieve his wakefulness, for several weeks before calling on me. As time passed, his sufferings became more and more severe. His facial expression changed, and in his whole appearance he was much different from what he had been the year before. He now complained of dimness of vision, and found it difficult to read his paper or write up his books. A distressing *tinnitus aurium* developed itself, and was especially annoying at night. His feet would "go to sleep" almost as soon as he took his seat in a chair, and he noticed that this symptom came first in the right foot, and persisted there after it had disappeared from the left leg. In attempting to write for any length of time he was compelled to stand, and of late he has noticed a sensation of pricking and tingling in the ring and little finger of the right hand when he has been working steadily with his pen. A sensation of extreme cold in the outer part of his right leg, extending from just below the knee to the ankle, developed itself a short time since and has caused him great discomfort. His walk is less secure than formerly, and his right foot has a tendency to drag. His handwriting has undergone a change, and, from being a bold, firm style, is loose and straggling, and occasionally illegible. His friends notice that in conversation he has a tendency to use wrong words, generally those having a sound similar to the ones he should employ, and that his voice is altered and his manner of speaking different from what it was before he became unwell.

During the first interview I had with him, he related many of the symptoms just detailed, but was disposed to ascribe most of his difficulties to disordered digestion—a condition which manifested itself very shortly after his business troubles commenced. His appetite was capricious, and an article which he had longed for most ardently would excite a sensation of disgust as soon as it was brought into his presence. Pain in the pit of the stomach and flatulence were especially distressing, and were present to a greater or less degree at all times. His bowels continued regular, and nothing abnormal was noticed about the renal secretion.

At the time he came under my care he was about 35 years of age. He could give a clear and concise family history for three generations, and numbered among his ancestors three persons who had been insane, one who had died of apoplexy, and one who during the past five years has been a helpless paralytic. His own health has always been good, and with the exception of an occasional attack of sudden painless vomiting, sometimes attended with profuse watery evacuations, he has never been sick.

The æsthesiometer revealed extensive and well-marked anæsthesia in various parts of the body. As compared with the corresponding situation on the other side, the anterior surface of the left forearm was hyperæsthetic, while this state of facts was reversed with regard to the left hand and arm. Well-marked anæsthesia could be demonstrated all over the right side, with the exception just noted. The patient stated that it took him some time to determine in his own mind, whether an object was or was not in contact with the right side of the body, while no such difficulty existed on the left side—a fact which was very apparent when he was tested to determine the point. In addition to his diminished sensibility to touch and contact, it was found that his sensibility to changes of temperature was likewise impaired, and that he was not competent to decide with his right hand the difference between two substances, one of which was 20° hotter than the other. Painful impressions were felt as acutely on the right as on the left side of the body, and the electro-muscular sensibility was not implicated on either side.

The muscular power, as denoted by the dynamometer, was very markedly diminished on the right side. With the left hand, he could mark 28°, while, despite his utmost exertions, he could barely reach 15° with his right. His grip was very uncertain, at one moment strong and another weak—that peculiar vacillating contraction of the muscles of the forearm which is characterized by the irregular and descending line traced by the dynamograph. The electro-muscular contractility was alike on both sides. The muscles were not atrophied to any appreciable extent, but seemed to be in a very fair condition.

The ophthalmoscope was next resorted to, and the following peculiar and characteristic appearances were noted at the back of the eye. The optic disk and its immediate neighborhood was obscured by a grayish-white coloration, and the appearances were such as to indicate an infiltration and swelling of the part. Immediately on the outside of this was a broad white band, which contrasted strongly with the red reflection of the fundus, and at the periphery of this zone a number of very small, but perfectly white, isolated spots could be discovered. In the region of the macula lutea these small white spots were numerous, and assumed a circular arrangement. Several traces of extravasated blood could be seen. The vascular supply of the disk was concealed from view; that of the retina was but little changed from a healthy state. The veins were slightly enlarged and a trifle more œdematous than normal; the arteries retained their usual dimensions.

At my request, a specimen of his urine was carefully examined by my friend Dr. Rankin, but aside from a slight excess of urates it was perfectly healthy. Another specimen was procured, which I examined, but could discover nothing in addition to what Dr. Rankin had found. For a number of weeks subsequently, the urine was tested every few days, but none of the evidences of kidney disease could be detected.

This gentleman traveled South in the latter part of December, and spent six or eight weeks in Havana. This trip had the effect of restoring his health in great measure, and upon his return, last February, he was in excellent spirits, and said that he had not been feeling so well for a year. An examination of his urine now revealed granular and hyaline casts, with a small amount of albumen. The ophthalmoscopic appearances were much less marked and striking than they were before, but were none the less characteristic. The broad white zone surrounding the parts in the immediate vicinity of the optic disk had disappeared, and was replaced by a structure of a slightly darker tint than the rest of the fundus; the edges of the disk which before were obscured could now be discerned with ease, while the discal vessels could be observed without difficulty. The circular arrangement of white spots previously seen at the macula lutea was unaffected. A number of white streaks had been developed along the course of some of the vessels. The spots of extravasated blood had all disappeared. A certain amount of difficulty of vision still remained.

This case does not stand alone, for my notes show several others in which the essential particulars—the degeneration of the retina and other ophthalmoscopic appearances of Bright's disease—have been discovered upon examination, without there being any rational symptoms or other physical signs of renal disease, yet in which subsequently the patients have developed albuminuria. It is but a few days since that I saw a typical example of this peculiar form of retinitis in a patient under the care of my friend, Dr. James F. Ferguson, with whom I saw the case in consultation, but no evidences of albumen or casts could be found, although the urine was separately examined by two different observers. Such cases are far from being common—at least so far as their recognition is concerned—yet their importance from a pathological and therapeutical point of view is such that they merit careful attention and study.

The case above is one that would ordinarily be accepted as a typical example of that form of cerebral disorder designated "congestion of the brain." The manner of its development—following prolonged and unusual mental anxiety and distress; its occurrence in an individual strongly predisposed to nervous disease; the symptoms complained of—insomnia, vertigo and headache, with hallucinations of the special senses of sight and hearing, and "referred sensations," such as the pricking and tingling in his extremities, and the feeling of coldness in his right leg; the mental depression and characteristic changes in speaking and writing; and the alterations of peripheral sensibility as determined by the æsthesiometer, and of muscular power, denoted by his manner of walking and by the use of the dynamometer—as also the striking fact that these last phenomena were more marked on one side than on the other—make a series of facts which point to the brain as the part at fault, and which indicate with almost certainty that the condition there existing is due to exhaustion of that organ. When this state of exhaustion is due to overwork of that part, it is generally, but not invariably, accompanied by overfulness of the cerebral vessels. It was for the purpose of determining the state of the cerebral circulation that the ophthalmoscopic examination was made, and I was surprised to discover that the brain symptoms which were so strongly marked, were merely symptomatic of Bright's disease, and were not due to primary disorders, either organic or functional, of the intra-cranial organs.

The peculiar form of vomiting complained of by this gentleman was the only symptom which can be considered to indicate in the slightest degree any renal disorder.

The connection between the retinitis and the organic changes in the kidneys is doubtless due to the operation of some one cause which produces them both. In other words, the degeneration of the retina and the degeneration of the kidneys are the common expression of some bodily condition which we are as yet unable to understand. When we consider the extremely delicate tissues of the former, and the comparatively stable structures of the latter, we cannot be surprised that the retina should present morbid changes before we can obtain any evidence of implication of the kidneys—the only wonder being that this mode of development should not be more common than the recorded observations would lead us to infer.

I am inclined to believe that a careful and systematic examination of the intraocular structures with the ophthalmoscope in every case in which nervous symptoms are complained of—these examinations being frequently repeated, and the patient watched for a length of time—will ultimately establish the fact that in a large proportion of cases Bright's disease will first manifest itself by the characteristic changes which it produces in the retina, and that these retinal appearances will be present for a length of time before any abnormal ingredients can be detected in the urine.

